# Epidemiologic and Economic Burden of Influenza in the Outpatient Setting: A Prospective Study in a Subtropical Area of China

**DOI:** 10.1371/journal.pone.0041403

**Published:** 2012-07-20

**Authors:** Ru-ning Guo, Hui-zhen Zheng, Li-qun Huang, Yong Zhou, Xin Zhang, Chan-kun Liang, Jin-yan Lin, Jian-feng He, Jin-qing Zhang

**Affiliations:** 1 Institute of Infectious Disease Prevention and Control, Center for Disease Control and Prevention of Guangdong Province, Guangzhou, China; 2 Zhuhai Municipal Center for Disease Control and Prevention, Zhuhai, China; 3 Institute of Pathogenic Microorganisms, Center for Disease Control and Prevention of Guangdong Province, Guangzhou, China; 4 Center for Disease Control and Prevention of Guangdong Province, Guangzhou, China; University of Hong Kong, Hong Kong

## Abstract

**Objectives:**

To understand the incidence of outpatient influenza cases in a subtropical area of China and the associated economic burden on patients' families.

**Methods:**

A hospital-based prospective study was conducted in Zhuhai City during 2008–2009. All outpatient influenza-like illness (ILI) cases were identified in 28 sentinel hospitals. A representative sample of throat swabs from ILI cases were collected for virus isolation using Madin-Darby canine kidney cells. The incidence of outpatient influenza cases in Zhuhai was estimated on the basis of the number of influenza patients detected by the sentinel sites. A telephone survey on the direct costs associated with illness was conducted as a follow-up.

**Results:**

The incidence of influenza was estimated to be 4.1 per 1,000 population in 2008 and 19.2 per 1,000 population in 2009. Children aged <5 years were the most-affected population, suffering from influenza at the highest rates (34.3 per 1,000 population in 2008 and 95.3 per 1,000 population in 2009). A high incidence of 29.2–40.9 per 1000 population was also seen in young people aged 5–24 years in 2009. ILI activity and influenza virus isolations adopted a consistent seasonal pattern, with a summer peak in July 2008 and the longest epidemic period lasting from July–December 2009. The medical costs per episode of influenza among urban patients were higher than those for rural patients. A total of $1.1 million in direct economic losses were estimated to be associated with outpatient influenza during 2008–2009 in Zhuhai community.

**Conclusions:**

Influenza attacks children aged <5 years in greater proportions than children in other age groups. Seasonal influenza 2008 and Pandemic influenza A (H1N1) 2009 had different epidemiological and etiological characteristics. Direct costs (mostly medical costs) impose an enormous burden on the patient family. Vaccination strategies for high-risk groups need to be further strengthened.

## Introduction

Extensive studies on influenza morbidity, hospitalization, and death have been performed in developed countries and regions, generating a consensus that influenza does bring greater health and economic burdens to the community [1]–[6]. Children aged less than 5 years are attacked by influenza in greater proportions than are children in other age groups [7]–[10]. In developing countries, especially in most tropical and subtropical low-income countries, less study on the burdens generated by influenza has been completed. Laboratory diagnostic detection for influenza is rarely carried out clinically, and there is often a lack of a comprehensive surveillance and evaluation system. Thus, the development and promotion of prevention and control strategies (such as vaccination strategies for high-risk populations) are accordingly limited in these areas.

No systematic study on the disease burden of influenza has been performed to date in China. Through the Computer Assisted Telephone Interviewing System (CATI), Guo et al. explored ILI morbidity in an urban community [11]. Zhang et al. evaluated the financial burden caused by the influenza pandemic in 2009 [12], but the disease burden of laboratory-diagnosed influenza in the community is still scarcely known. Guangdong province is located in the subtropical area of south China, a location once hypothesized to be the epicenter of pandemic influenza [13]. In the 1970s, Guangdong started influenza surveillance, and it joined the World Health Organization (WHO) Global Influenza Surveillance Network in 1998. For years, Guangdong has sent influenza virus-positive specimens to the Chinese National Influenza Centre, which sends viral isolates to the WHO. Specimens from Guangdong province represent a significant proportion of the viral isolates that China has shared with the WHO Global Influenza Surveillance Network. Considering the important position of Guangdong in influenza surveillance, a Collaboration Center for Surveillance and Research on Emerging Infectious Diseases was established in 2005 between Guangdong and WHO.

With the support of WHO, a project to evaluate the ILI and influenza burden in Guangdong was launched in 2006. The research group first completed a population-based household survey on the ILI burden in 2007 in Zhuhai prefecture, accumulating some meaningful findings on the incidence and economic burden of ILI [14]–[15]. To understand laboratory-confirmed influenza cases and their associated costs to patients' families, a prospective study on the outpatient influenza burden was conducted in the same community during 2008–2009. It is worth mentioning that influenza illness in China is often associated with health care being sought in an outpatient setting [11], [12], and patients with severe symptoms who may need to be hospitalized are first detected in outpatient departments as well. Therefore, outpatient data with standardized ILI registration and reporting processes can largely reflect the intake of influenza cases to hospitals.

As such, the aim of this study is to grasp the epidemiological and etiological characteristics of outpatient influenza cases and the associated medical and other direct costs paid by patients; the goal is ultimately to help in understanding the incidence of outpatient influenza cases and the associated economic burden on people in subtropical areas of China.

## Materials and Methods

### Study community and population

Given such factors as its population size, the number of medical institutions within its jurisdiction, and its routine use as a base for ILI surveillance over the years, Zhuhai City was selected as the sentinel community for the study on influenza burden. With an area of 1,952 square kilometers and a jurisdiction including 3 districts 15 towns, Zhuhai has a resident population of 1.37 million; 0.32 million (23.1%) are 0–14 years old, 0.98 million (71.7%) are 15–64 years old, and 0.72 million (5.2%) are over 65 years old. Our sentinel sites include 28 hospitals (20 public hospitals and 8 community/school clinics) with a collective catchment of over 80% of the total population of Zhuhai, according to a survey of the situations of medical institutions in Guangdong in 2009.

All outpatients at sentinel sites who met the criteria for ILI during 2008–2009 were included in our study. ILI is defined as sudden onset of fever (38°C or above) with cough, sore throat, or other respiratory symptoms. A laboratory-confirmed influenza case is determined to be positive for influenza virus by isolation from culture. An investigation of the economic burden of ILI cases was conducted from June 2008 to May 2009; a total of 1,453 cases of ILI were included in the survey, 153 of which became laboratory-confirmed influenza cases.

### Data, collection, and specimen detection

Sentinel hospitals registered ILI patients in the outpatient departments of respiratory medicine, pediatrics, emergency medicine, infectious diseases, and traditional Chinese medicine; the results were summarized weekly and reported monthly to the provincial CDC.

A representative number of throat swab specimens from ILI cases were collected. With the average 10% virus-positive rate among ILI patients in Guangdong over the years, an allowable error of 15%, and a significance level of 0.05, a sample size of 1,600 throat swabs was predicted to be necessary by the simple random sampling method. The community needed to provide at least 1,600 throat swabs per year, i.e., approximately 30 per week. Taking into account the convenience of the actual work, hospitals randomly selected 1 or 2 days (Wednesday/Thursday) per week, and all ILI cases on that day were included for case investigation and specimen collection. Oral consent was obtained from each ILI patient. Specimens were quickly preserved in Hank's sample media at pH 7.4–7.6 (adding 0.1 mg/ml gentamicin and 2 µg/ml antifungal factors), then transported to Zhuhai CDC laboratories at 4°C within 24 hours. Specimens were stored at −70°C until they could be tested for virus isolation in Madin-Darby canine kidney (MDCK) cells. The Zhuhai CDC submitted influenza isolates to the provincial CDC for subtyping using the hemagglutination inhibition assay. Virus isolation and hemagglutination inhibition assays were performed according to the World Health Organization Manual on Animal Influenza Diagnosis and Surveillance [16].

**Figure 1:**
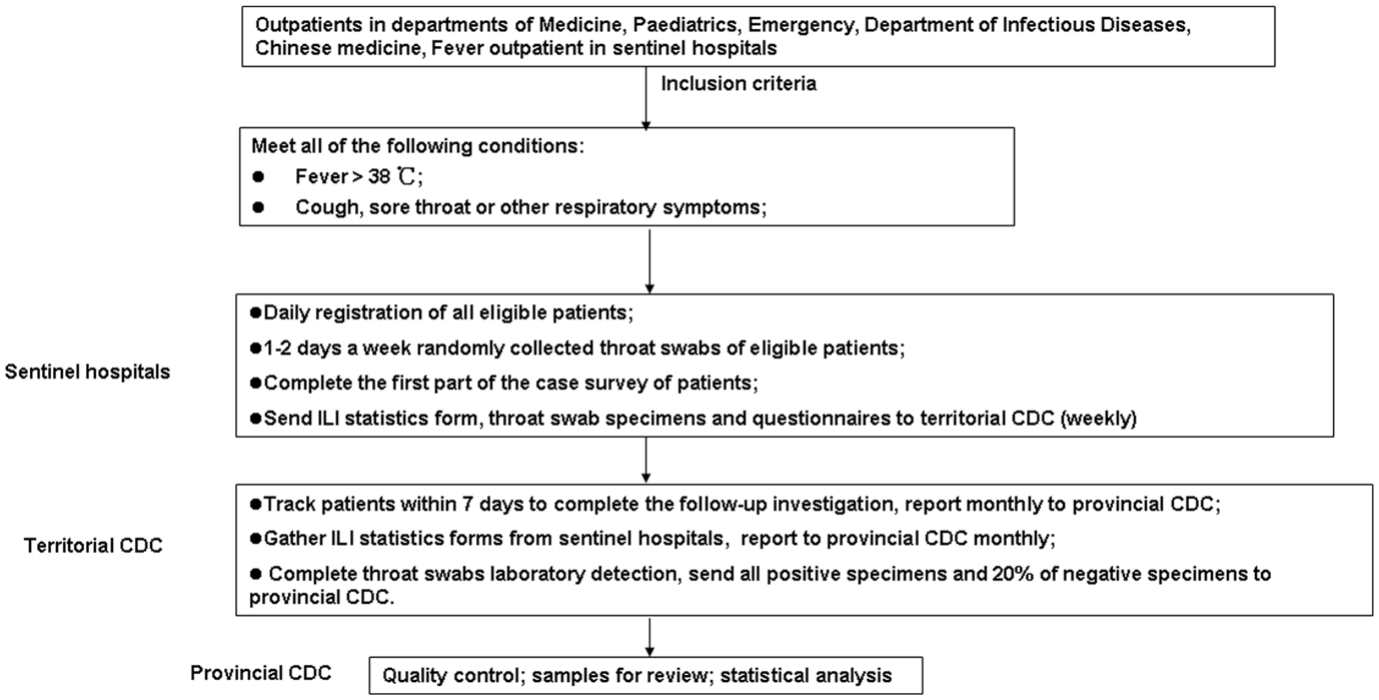
Data and specimen collection procedure. Twenty-eight hospitals in Zhuhai City were selected for a sentinel Influenza-Like Illness (ILI) and influenza survey. All of the sentinel sites' outpatient ILI cases fulfilling the inclusion criteria were identified. A random sample of throat swabs from ILI cases were collected for influenza virus isolation at the territorial CDC. An economic burden survey was completed by ILI patients whose throat swabs were collected within 7 days after their specimens were collected.

A survey of economic burden was conducted on ILI patients whose throat swabs were collected. A structured questionnaire was designed, including patients' demographic information, clinical course, medical consultation expenses, and other direct costs associated with this disease (transportation, additional food, treatment in other hospitals, OTC drugs, room and board, etc.). Investigators have medical backgrounds and received uniform training prior to the survey. Follow-up investigation was completed via telephone call within 7 days after specimens were collected. Below is the data and specimen collection procedure (Figure 1).

### Incidence projections

The number of ILI cases in sentinel hospitals was obtained through ILI registration. The throat swab specimens collected in ILI cases were analyzed for virus isolation. We obtained the age-specific isolation rates for influenza virus by which to estimate the age-specific numbers of outpatient influenza cases in the sentinel sites.

The incidence of outpatient influenza cases in Zhuhai was estimated on the basis of the number of outpatient influenza cases detected by the sentinel sites, considering the proportion of the census population who would seek care outside of the sites' catchment area. As described before, the catchment area of our study hospitals covers 80% of the residents in the entire city. We could estimate the total and age-specific number of outpatient influenza cases in Zhuhai community by multiplying number of outpatient influenza cases in sentinel hospitals by 1.25 in order to obtain the average and age-specific rates of outpatient influenza in the community using demographic data.

### Direct economic burden of influenza

The direct cost of influenza is defined as the total cost of consultation, including medical expenditures and other direct costs associated with the illness. Other direct costs result from OTC drugs, treatment other than this hospital visits (probably multiple consultations), additional food, transportation, and room and board. Some measurement standards were unified, such as transportation costs (20 RMB [$2.92] round trip for car and 6 RMB [$0.88] for motorcycle).

### Ethics Statement

Collecting throat swab of Influenza-like illness has become a routine method in diagnosis of influenza clinically. Throat swab specimen collection process and the impact on the subjects is extremely small, with the risk no greater than for routine physical or psychological examination of subjects. Case investigation will not reveal the patient's personal information, privacy and confidentiality, and patients' rights or interests will not be violated. Under the premise of ensuring no violation of “Helsinki Declaration” on the provisions of human subjects, with simple and operable principle, the ethical review committees of Centers for Disease Control and Prevention of Guangdong Province agreed the implementation of throat swab specimens collection and case investigation via oral informed consent form, and oral informed consent was obtained from each participant who was randomly selected for case survey and throat swab collection.

## Results

### Epidemiological characteristics of ILI

A total of 40,894 ILI cases were detected in 2008, accounting for 3.27% of total outpatient cases (40,894/1,251,395). Children aged <5 years constituted 58.5% of the total number of ILI cases, followed by the 5–15-year-old age group, which constituted 19.1% of cases. The number of ILI cases in all age groups followed a similar seasonal pattern: it formed a March–August epidemic wave with a peak in July.

**Figure 2:**
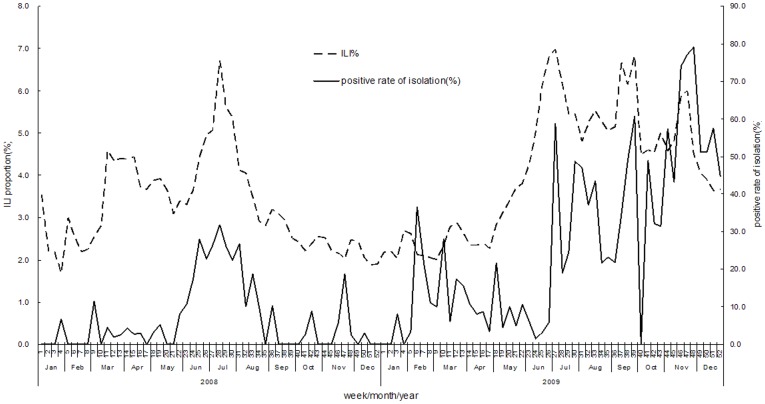
Proportion of Influenza-Like Illness (ILI) cases of total outpatients and influenza virus isolation rate by week. ILI activity followed a similar seasonal pattern to the rate of influenza virus isolation in 2008–2009. A summer peak in July occurred in 2008, while a different epidemic pattern happened in 2009. In 2009, The proportion of outpatients with ILI and virus isolation rates increased significantly from week 21 to week 52, resulting a long epidemic period during which 3 peaks occurred (in weeks 27, 39, and 47).

In 2009, 74,242 cases of ILI were detected, accounting for 4.25% of total outpatient cases (74,242/1,745,899). Children aged <5 years occupied 40.4% of the total number of ILI cases, followed by the 5–15-year-old age group, which constituted 24.4% of cases. The number of ILI cases in 2009 assumed a seasonal pattern distinct from that observed in 2008 and past years. The number of ILI cases increased significantly from week 21 until week 52, resulting in a long epidemic period, during which 3 peaks occurred in weeks 27, 39, and 47 (Figure 2).

### Etiological characteristics

**Figure 3:**
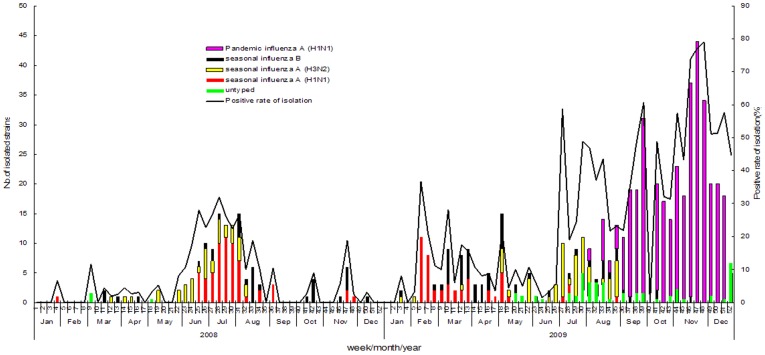
Influenza virus isolations among outpatient Influenza-Like Illness (ILI) cases. Seasonal influenza 2008 and Pandemic influenza A (H1N1) 2009 had different etiological characteristics. In 2008, the highest isolation rate occurred in June–August, with seasonal influenzas A (H1N1) and A (H3N2) as the advantage strains (accounting for 45.9% and 27.4% of total isolates, respectively). Unlike the June–August peak in 2008, the first small peak of seasonal influenza appeared as early as February–April in 2009; after that, influenza activity slowed down in May–June, then increased beginning in July. The average positive virus isolation rate remained 45.5% in July–December, producing a long influenza virus activity period. The advantage strain in 2009 was pandemic influenza A (H1N1), comprising 59.3% of total isolates.

In total, 1,485 throat swabs from ILI cases were collected for virus detection in 2008, with a virus isolation rate of 9.1% (135/1,485). Isolation rates showed significant variation on the basis of age (Table 1). A higher isolation rate was seen in people aged over 15 years (over 20.0%), and the lowest rate was seen in children aged less than 5 years (7.2%). There was also a seasonal pattern: the highest virus isolation rate occurred in June–August (>20%) in weeks 25–31; the highest one-week rate (31.9%) was seen in week 28. One hundred thirty-five viral isolates were identified for genotyping, of which seasonal influenza A (H1N1), influenza A (H3N2), influenza B, and unclassified strains accounted for 45.9%, 27.4%, 23.7%, and 3.0% of total isolates, respectively. The advantage strain also changed by season (see Figure 3).

**Table 1:** Estimates of age-specific incidence rates of outpatient influenza in a prospective study in south China, 2008–2009.

Age group (years)		0–4	5–14	15–24	25–59	>60	Unknown	Total
2008	Mid-year population	62755	256154	226639	687484	126170	/	1359202
	Number of ILI cases in sentinel hospitals (i)	23943	7791	3710	4646	601	203*	40894
	Age-specific virus isolation rate of ILI (%)	7.2	8.6	20.5	25.4	20.0	/	9.1
	Number of outpatient influenza cases in sentinel hospitals (ii)	1724	670	761	1180	120	/	4455
	Estimated number of outpatient influenza cases in the community (iii)	2155	838	951	1475	150	/	5568
	Incidence of outpatient influenza cases (per 1,000 population)(iiii)	34.3	3.3	4.2	2.1	1.2	/	4.1
2009	Mid-year population	63066	253104	229789	694409	129151		1369519
	Number of ILI cases in sentinel hospitals (i)	30062	18173	14046	9871	2037	53*	74242
	Age-specific virus isolation rate of ILI (%)	16.0	45.6	38.2	24.2	8.3	/	28.2
	Number of outpatient influenza cases in the sentinel hospitals (ii)	4810	8287	5366	2389	169	/	21020
	Estimated number of outpatient influenza cases in the community (iii)	6012	10359	6707	2986	211	/	26275
	Incidence of outpatient influenza cases (per 1,000 population)(iiii)	95.3	40.9	29.2	4.3	1.6	/	19.2

“i” is the number of influenza-like illness (ILI) patients in the sentinel hospitals. “ii” is the number of outpatient influenza cases in the sentinel hospitals. This number is obtained by multiplying “i” by the age-specific influenza isolation rate associated with ILI. The age-specific influenza isolation rate of ILI is obtained through laboratory detection for a representative random sample of ILI cases. “iii” is the estimated number of outpatient influenza cases in the community . It is obtained by correcting “ii” for a catchment area ratio of 80%. “iiii” is the incidence of outpatient influenza cases in the community. It is obtained by dividing “iii” by mid-year population.

* There were 203 and 53 ILI cases in 2008 and 2009, respectively, whose age registration information was lost.

“/” means that no case counting or analysis was done on corresponding variables.

In 2009, a total of 2,144 throat swabs from ILI cases were collected, with a virus isolation rate of 28.2% (604/2,144), well above the historical average level. The highest isolation rate (45.6%) was seen in people aged 5–14 years, followed by 38.2% in people aged 15–24 years and 24.2% in people aged 25–59 years. The lowest virus isolation rate (8.3%) occurred in people aged over 60 years. In contrast with the June–August peak in 2008, 2009's first small peak of seasonal influenza appeared as early as in February–April; after that, influenza activity slowed down in May–June, it increased again beginning in July. The average positive isolation rate remained at 45.5% in July–December and reached its highest one-month rate (66.2%) in November; the highest one-week rate (79.1%) was observed in week 48. Of the 604 viral isolates, seasonal influenza A (H1N1) and A (H3N2) viruses accounted for 9.3% and 10.3% of influenza isolates, respectively; influenza B virus accounted for 9.1%; pandemic influenza A (H1N1) accounted for 59.3%; and another 12.1% of samples were unclassified.

The etiological characteristics of influenza in 2009 were significantly different from those in 2008 and past years. The main strain of influenza was seasonal influenza A (H1N1) virus in January–February (accounted for 84.0%). Influenza B virus and seasonal influenza A (H1N1) combined to account for the vast majority of isolates in March–April (60.5% and 37.2% , respectively). Beginning in May, seasonal influenza A (H3N2) virus increased in prevalence and became the dominant strain in May–July (46.6%), during which time the percentage of isolates with unclassified strains increased markedly (25.0%). In August, isolation of Pandemic influenza A (H1N1) 2009 began and increased, promptly becoming the absolute advantage strain in September–December (comprised 89.8% of isolates; see Figure 3).

### Estimation of incidence of influenza

A total of 4,455 outpatient laboratory-confirmed influenza cases were identified in sentinel sites in 2008. Assuming that the catchment area of the sentinel sites included 80% of the population of the area, the number of outpatient influenza cases in the community was estimated to be 5,568, by which the incidence of outpatient influenza was inferred to be 4.1 cases per 1,000 population. Children aged <5 years had the highest incidence (34.3 per 1,000 population; see Table 1).

In 2009, a total of 21,020 influenza cases were identified in sentinel hospitals, and 26,275 cases of outpatient influenza were estimated in the community, by which the incidence of outpatient influenza was inferred to be 19.2 cases per 1,000 population in 2009. Children aged <5 years had the highest incidence at 95.3 per 1,000 population, followed by those aged 5–14 years at 40.9 per 1,000 population, those aged 15–24 years at 29.2 per 1,000 population, and those aged >60 years at 1.6 per 1,000 population (see Table 1).

### Direct economic burden of influenza outpatients

One thousand four hundred fifty-three ILI cases were included in the economic burden survey, and 153 of those were laboratory-confirmed influenza patients. The economic burden survey showed that the average medical expense per episode of influenza was $22.8 (95% confidence interval: $20.9–24.8). Patients living in urban areas incurred higher costs than patients in rural areas. Costs per episode of influenza were $25.5 ($23.1–27.9) for urban patients and $17.4 ($14.69–20.3) for rural patients (see Table 2).

**Table 2:** Direct economic burden analysis of 153 cases of confirmed influenza from a prospective influenza burden study in south China, 2008–2009.

Cost (Yuan)*	Rural patients				Urban patients			
	Mean	95% confidence interval for mean	Minimum	Maximum	Mean	95% confidence interval for mean	Minimum	Maximum
Medical expenses	119.5	99.9–139.1	10	290	174.6	158.1–191.1	18	500
Over-The-Counter (OTC) drugs	1.1	0.45–2.61	0	30	1.9	0.26–3.5	0	50
Treatment costs other than this hospital visit	31.1	14.3–48.0	0	205	26.7	15.1–38.3	0	300
Transportation	5.2	2.6–7.9	0	40	8.7	6.1–11.2	0	50
Additional food	5	0.9–10.9	0	100	3.9	0.5–7.3	0	100
Room and board	15	2.1–27.9	0	200	23.6	12.6–34.6	0	300
Total	178	140.8–215.2	10	544	239.3	214.1–264.6	22	820

* The Yuan is the Chinese currency. 1 Yuan = USD 0.146.

Other direct costs incurred included transportation, additional food, Over-the-Counter (OTC) drugs, consultation in other hospitals, etc. The results showed that only 5.9% of patients spent about $3.0–$7.3 on OTC drugs, and 28.8% of patients had more than $14.6 in medical costs before the consultation completed for the study. The direct costs associated with one episode of influenza were about $34.9 and $26.0 for urban and rural patients, respectively.

These results enable us to render a preliminary estimation of the total direct costs by outpatient influenza in 2008–2009. As previously projected, our whole community had 5,568 and 26,275 cases of laboratory-confirmed influenza in the outpatient setting in 2008 and 2009, respectively; that is, patients needed to spend $1.1 million (about $0.2 million in 2008 and $0.9 million in 2009) on direct economic costs associated with outpatient care.

## Discussion

Our prospective hospital-based influenza burden study has explored the incidence and associated economic costs of laboratory-confirmed outpatient influenza for the first time in China. As the first attempt to probe the feasibility and operability of disease burden assessment in the Chinese community, our study has practical reference value for other regions in China and Tropical and Subtropical Regions to estimate the disease burden of influenza.

In southern China, seasonal influenzas A (H1N1), A (H3N2), and B are prevalent together, only alternating advantage strains by season. In 2009, an apparent delayed epidemic wave occurred and produced a long epidemic period. This abnormal feature was blamed during follow-up by pathogen monitoring on the occurrence of a major epidemic of Pandemic influenza A (H1N1), which was distinct from 2008 and past years.

Our results showed that ILI activity followed similar seasonal patterns with the positive isolation rate of influenza virus in 2008–2009. In 2008, both peaked in July, and in 2009, both rose sharply in July, then remained at high levels from August to December, producing a long epidemic period. These results indicate that local ILI syndrome surveillance could indirectly reflect the activity of laboratory-confirmed influenza.

The health burden imposed by influenza cannot be ignored. We estimated that the annual incidence rates of outpatient influenza were 4.1 per 1,000 population in 2008 and 19.2 per 1,000 population in 2009. A total of 5,568 and 26,275 outpatient influenza cases were estimated in the community in 2008 and 2009, respectively. Children less than 5 years old are the main targets of seasonal epidemics [7]–[9], [17]–[18], while pandemic influenza A (H1N1) 2009 in Zhuhai attacked a wider age group (less than 25 years old). These results convey practical evidence for the formulation of local influenza prevention and control strategies that target immunization strategies toward high-risk groups [19].

In addition to impacts on its large number of patients, influenza also produced significant pressure on patients' families. The direct economic costs (primarily medical costs) for one episode of influenza occupy 5–15% of the average monthly family income among local residents [20], directly leading to $1.1 million in economic losses. These figures are probably conservative, as our study includes only outpatients, whereas hospitalization often results in greater economic expenditures. A more comprehensive evaluation of influenza burden needs to be conducted in the future.

We may be underestimating the true disease burden of influenza among outpatients. The economic burden of influenza would be higher if we considered indirect burdens caused by absenteeism from work, escorting patients, and visiting costs. Furthermore, influenza patients who do not satisfy ILI criteria can also lead to underestimation of the influenza burden. In addition, information bias is produced when patient data is retrospectively collected. Usually, local CDC staff conducted a telephone follow-up investigation within 7 days after receiving the questionnaires. Factors such as memory and respondent compliance may affect the quality of the data, and investigators cannot simply judge the authenticity of information obtained by telephone.

Overall, this study provides the first use of a pilot community in south China to develop an influenza burden assessment system; this study will provide practical evidence for further studies of influenza burden in the future. Although this study has many inadequacies, our research has practical reference value for similar research in other districts of China and the Tropical and Subtropical Regions.
